# Relationship between inpatient satisfaction and nurse absenteeism: an exploratory study using WHO-PATH performance indicators in France

**DOI:** 10.1186/1756-0500-5-83

**Published:** 2012-01-31

**Authors:** Leila Moret, Emmanuelle Anthoine, Cécile Paillé, Sophie Tricaud-Vialle, Laurent Gerbaud, Alexandra Giraud-Roufast, Philippe Michel, Pierre Lombrail

**Affiliations:** 1 Public Health Department--PIMESP, University Hospital of Nantes, Hospital Saint-Jacques 85, rue Saint-Jacques, 44093 Nantes cedex, France; 2 Bordeaux University Hospital, Comité de Coordination de l'Evaluation Clinique et de la Qualité en Aquitaine (CCECQA), Pessac, France; 3 Clermont-Ferrand University Hospital, Groupe régional d'évaluation en Auvergne (GREQUAU), Clermont-Ferrand, France

## Abstract

**Background:**

Indicators describing results of care are widely explored in term of patient satisfaction (PS). Among factors explaining PS, human resources indicators have been studied in terms of burnout or job satisfaction among healthcare professionals. No research work has set out to explore the effect of absenteeism on PS scores. The objective of this study was to explore interaction between rate of absenteeism among nurses and PS results.

**Methods:**

France has taken part in a project named PATH (Performance Assessment Tool for Hospitals) of the World Health Organization, aiming to develop a tool for the assessment of hospital performance. In the first semester 2008, 25 volunteering short-stay hospitals (teaching, general and private) provide complete data on nurse short-absenteeism (periods of up to 7 consecutive days of sick leave) and on PS (a cross-sectional postal survey using a standardized validated French-language scale EQS-H exploring "quality of medical information" (MI) and "relationships with staff and daily routine" (RS)). A multi-level model was used to take into account of the hierarchical nature of the data.

**Results:**

Two thousand and sixty-five patients responded to the satisfaction questionnaire (participation rate: 40.9%). The mean age of respondents was 58 yrs (± 19), 41% were men. The mean duration of hospitalisation was 7.5 days (± 11.1). The mean absenteeism rate for nurses was 0.24% (± 0.14).

All the PS scores were significantly and negatively correlated with rate of short-absenteeism among nurses (MI score: *ρ *= -0.55, *p *< 0.01), RS score *ρ *= -0.47, *p *= 0.02). The mixed model found a significant relationship between rate of absenteeism among nurses and PS scores (MI: *p *= 0.027; RS: *p *= 0.017).

**Conclusion:**

Results obtained in this study show that short-term absenteeism among nurses seems to be significantly and negatively correlated with PS. Our findings are an invitation to deepen our understanding of the impact of human resources on PS and to develop more specific projects.

## Background

Improving the performance of healthcare facilities is a central theme for hospitals, whether for professionals or for users of the healthcare system. Performance is by essence multidimensional, since objectives and expectations are numerous and varied [1]. The conceptual models developed thus far, such as the PATH (Performance Assessment Tool for Hospitals) of the World Health Organization (WHO) [2], or the EFQM (European Foundation for Quality Management) health model [1,3], converge as to the main dimensions that should be taken into account, such as efficiency, clinical efficacy, safety, human resources and the patient-centered approach. The value of these models also resides in their integrated approach, arising from the interdependence among the different dimensions [2]. Among all these dimensions, interrelations between human resources and patient-centered approach dimensions are interesting to focus on. First of all, indicators of patient-centeredness dimension are usually characterized by patient satisfaction surveys, considered to be global outcome indicators in the evaluation of quality of care and hospital performance [4]. Most health care organizations are able to regularly collect user experiences and numerous studies exploring patient needs and expectations and determinants of patient satisfaction have been conducted. Most of this work has explored clinical characteristics and demographic factors, but findings on the impact of these factors on satisfaction scores are inconsistent and diverge from one study to another. According to the literature, patient age, subjective health status and satisfaction with life in general are the main predictors of satisfaction results [5-11].

Moreover, while two major dimensions are generally seen as to conditioning the quality of patient-caregiver interaction--the medical information delivered by caregivers and relationships with caregivers--only a few studies have explored relationships between patient satisfaction and atmosphere in the workplace or job satisfaction among healthcare professionals [12,13]. Vahey showed that burnout among nurses affects patient satisfaction with their care [13]. Several studies [14-16] have suggested that "magnet" hospitals have lower staff turnover and greater job satisfaction. Patient satisfaction seems to be greater when units have adequate staff and good administrative support for nursing care.

To our knowledge, no research work has set out to explore the effect of absenteeism among nurses on patient satisfaction scores. The aim of this study was to explore interactions between two performance dimensions of the OMS-PATH model: human resources and patient-centered approach. The objective was to explore relationships between rates of absenteeism among nurses and patient satisfaction results using a large sample of hospitals taking part in the OMS-PATH performance assessment project in France [17].

## Methods

### Study design

France has twice taken part in the WHO-PATH project. The first phase was implemented in 2004 and the second in 2007. The WHO-PATH conceptual model integrates 6 hospital performance dimensions: "clinical effectiveness", "efficiency", "human resources", "responsive governance", "safety" and "patient centeredness".

All the French hospital facilities participating in the PATH project that had data on these two indicators were included in the analysis. The sample comprised the 25 facilities that could provide complete data for these two dimensions out of the 48 short-stay participating hospitals (teaching, general and privately managed facilities) included in a voluntary basis. These 25 hospitals seemed to have the same key characteristics that the others in terms of number of beds (median was 138 [38-1670] versus 122 [15-689]--*p *= 0.04, type of facility (public or private) (*p *> 0.05) and region of origin (*p *> 0.05)).

### Satisfaction indicators for hospitalised patients

According to OMS-PATH project, a retrospective cross-sectional postal survey was implemented on a sample of 100-250 patients per hospital facility according to size; all patients complying with inclusion criteria received a questionnaire, as they were included.

The patients included had left the facility 2 weeks to 1 month preceding the dispatch of the letter. The evaluation concerned the last hospital facility frequented by the patient. Questionnaires were sent out in February and March 2008. The patients completed the questionnaires and returned them directly to the PATH coordination unit in Nantes teaching hospital for data capture and analysis.

#### Inclusion criteria

• Hospitalisation for at least 2 consecutive nights in adult short-stay facility (medicine, surgery, obstetrics) concerning patients of 18 or over

• Patients resident in France, and returning directly to their usual place of residence on discharge from the short stay unit, including homes for the elderly

#### Exclusion criteria

• Patients hospitalised for less than 2 consecutive nights in a short-stay facility, or hospitalised in rehabilitation, long-term care or psychiatry units

• Patients transferred to another facility, or patients who died during hospitalisation

• Patients hospitalised anonymously or confidentially, or homeless individuals

• Patients declining to respond to the questionnaire or unable to do so

#### Satisfaction indicators

The three satisfaction scores for hospitalised patients belonging to the French-language scale EQS-H ("Echelle de Qualité des Soins en Hospitalisation"), validated in the literature, were calculated [18]:

• a satisfaction score relating to quality of medical information (MI)

• a satisfaction score relating to relationships with staff and daily routine (RS)

• a global satisfaction score

The EQS-H scale comprises 16 items divided between two dimensions: MI (8 items) and RS (8 items). The validation study on this scale showed excellent validity and stability [18]: the first two factors explained 66% of the variance, and Cronbach's alpha coefficient for the overall scale was 0.95. A confirmation study was conducted, and similar psychometric properties were found: the two factors explained 67% of the variance and Cronbach's alpha coefficient for the overall scale was 0.93.

Five response choices are provided: "poor", "average", "good", "very good" and "excellent". Scores are attributed to each response choice (0-25-50-75-100), with higher values corresponding to greater satisfaction. Individual scores are calculated for all patients who responded to at least half the items plus one in a dimension. The scores are calculated by summing responses to items and then dividing by the number of items completed. The mean score of a dimension is the sum of individual scores divided by the number of respondents concerned. Satisfaction scores range from 0 to 100.

The patient characteristics used as adjustment variables for the scores were age with a threshold at 65 years, gender, and general satisfaction with life, coded from 1 to 7 [9,19].

### Indicators for absenteeism among nurses

Absenteeism corresponded to failure by staff to present in accordance with planned duty hours. The rate of absenteeism of short-term was defined by the WHO-PATH project as the sum of days off work on medical grounds, relating to periods of up to 7 consecutive days of sick leave, multiplied by 100, and divided by the number of equivalent full-time posts on the payroll, multiplied by 365 days. The period of the study was the first semester 2008.

One-day absences without justification were not counted, nor were days of absence for vacation and other forms of special leave, for training courses or other absences for professional reasons, or for maternity leave.

All qualified nurses in permanent posts were included.

### Characteristics of the hospital facilities

The descriptive characteristics of the hospital facilities were their size, described in terms of the number of beds in medicine, surgery and obstetrics, the type of structure: public or privately managed, their French regions, their absenteeism rates and their training expenditure rates (training expenditure among all hospital facility expenditure).

### Statistical analysis

#### Univariate descriptive statistics

To describe the characteristics of the sample, frequencies, means, standard deviation and range were calculated. Because of the small number of hospital facilities involved and the absence of normality in distributions, non-parametric statistics (Spearman's correlation test and Wilcoxon's means comparison test) were used for results according to facility (n = 25).

#### Multivariate statistics: multi-level model

The patient observations were grouped into clusters of hospital facilities. A multi-level model was constructed to take account of this hierarchical data structure and the hospital facility effect.

#### Relationship between patient satisfaction and absenteeism among nurses

Short-term absenteeism among nurses was significantly correlated with the number of hospital beds and with the type of facility. The qualitative variable "public or privately-managed facility" was included in the model, as well as three patient characteristics: age with a threshold at 65 yrs, gender, and satisfaction score for life in general (median threshold: 4).

Individual level equation for patient i in hospital facility j

Hospital facility level equations

Scoreij is the value of the score patient i in facility j among the 25 hospital facilities having taken part in the satisfaction survey and having collected data on absenteeism among nurses.

βagei is regression coefficient of the age fixed effect at the individual level.

x_agei*j *_is the fixed effect variable for patient i in hospital facility j at the individual level.

r_*ij *_is the error for patient i in hospital facility j at the individual level.

β_absenteeism _is the regression coefficient of the absenteeism fixed effect at the hospital facility level, it is identical for all groups.

x_absenteeism*j *_is the fixed effect variable in hospital facility j at the hospital facility level.

u_0*j *_is the error in hospital facility j at the hospital facility level.

The significance threshold is set at 5%.

The data analysis was performed on S-PLUS 6.0 and R 2.9.0 software.

## Results

### Profile of the 25 hospital facilities

The facilities were evenly distributed between public and privately managed establishments (Table 1). The number of beds in medicine and surgery was significantly greater in the public facilities (*p *< 0.01). Six hospitals facilities were in Pays de la Loire, 3 were in Auvergne, 14 were in Aquitaine and 2 in 2 other French regions.

**Table 1:** Profiles of hospital facilities

	Type of facility	
	**Public**	**Private**

Number of facilities	13	12

Median n° of beds [range]	357 [79-1670]	99 [38-268]

Median number of nurses [range]	576.4 [77-2211.6]	55.5 [24-183.9]

Mean absenteeism rate (± SD)	0.33 (± 0.12)	0.15 (± 0.10)

Mean training expenditure rate (± SD)	2.90 (± 1.3)	2.1 (± 1.1)

### Description of absenteeism

The mean absenteeism rate for nurses in hospital facilities was 0.24% (± 0.14). This rate in the privately managed facilities was significantly below that for public facilities (*p *< 0.001) (Table 1). In addition, there was a significant positive correlation between absenteeism among nurses and the number of beds in medicine and surgery in the facilities (*ρ *= 0.55; *p *< 0.001).

### Profiles of respondent patients and satisfaction scores

In the 25 hospital facilities taking part, 2065 patients responded to the satisfaction questionnaire (out of 5050 dispatched) giving a response rate of 40.9%.

The mean age of respondents was 58 yrs (± 19) and the median age was 60. Forty-one percent (n = 846) of respondents were men. Two thirds had undergone surgery (n = 1363) in the course of their hospitalisation, and 36% had been admitted in emergency (n = 743). The mean duration of hospitalisation was 7.5 days (± 11.1).

The mean participation rate for the satisfaction survey was not very different in public (35.7%) and private facilities (33.1%) (*p *= 0.41).

The satisfaction scores, however, were significantly higher in private hospital facilities than in public facilities (Table 2). In addition, MI score (*ρ *= -0.13; *p *< 0.01), RS score (*ρ *= -0.12; *p *< 0.01) and the overall score on the EQS-H scale (*ρ *= -0.12; *p *< 0.01) were significantly and negatively correlated with the number of beds in medicine and surgery units.

**Table 2:** Satisfaction scores by type of facility

	Public facility		Privately managed facility		
***Scores***	**Mean ± SD**	**Numbers**	**Mean ± SD**	**Numbers**	***p*-value**

MI* score	58.1 ± 23.1	987	62.6 ± 21.6	893	< 0.001

RS** score	68.6 ± 19.8	1052	71.5 ± 19.3	945	< 0.001

Global Score	63.7 ± 20.3	978	67.2 ± 19.2	879	< 0.001

* Quality of medical information

** Relationships with staff and daily routine

### Relationship between absenteeism among nurses and patient satisfaction

MI score (*ρ *= -0.55; *p *< 0.01), RS score (*ρ *= -0.47; *p *= 0.02) and the overall score on the EQS-H scale (*ρ *= -0.54; *p *< 0.01) were significantly and negatively correlated with short-absenteeism among nurses.

The mixed model made it possible to show that absenteeism among nurses significantly explained the variance of the patient satisfaction scores, after adjustment for the type of facility (public or private) and patient characteristics. This relationship was verified for the MI score (β = -19.76 [-37.14;-2.39]; *p *= 0.027), the RS score (β = -20.20 [-36.53;-3.87]; *p *= 0.017) and for the overall score on the EQS-H scale (β = -20.24 [-36.17;-4.32]; *p *= 0.015) (Table 3).

**Table 3:** Relationship between absenteeism among nurses and patient satisfaction

EQS-H score		b	*CI95%	*p*-value
**MI score** n = 1791	*Fixed*			
	
	Intercept	54.51	[47.96; 61.06]	< 0.001
	
	Absenteeism	-19.76	[-37.14; -2.39]	**0.027**
	
	Hospital facility			
	
	Public	Ref		
	
	Private	0.66	[-4.16; 5.48]	0.780
	
	Age of patient			
	
	< = 65 yrs	Ref		
	
	> 65 yrs	-3.39	[-5.62; -1.15]	0.003
	
	Patient gender			
	
	Female	Ref		
	
	Male	3.59	[1.43; 5.74]	0.001
	
	Satisfaction with life			
	
	< = 4	Ref		
	
	> 4	12.11	[9.29; 14.94]	< 0.001
	
				
	
	*Random*	σ_b_(between variability)	CI95%	
	
	Facility	3.28	[2.05; 5.25]	

**RS score** n = 1900	*Fixed*			
	
	Intercept	67.61	[61.56; 73.67]	< 0.001
	
	Absenteeism	-20.20	[-36.53; -3.87]	0.017
	
	Hospital facility			
	
	Public	Ref		
	
	Private	-1.01	[-5.56; 3.53]	0.647
	
	Age of patient			
	
	< = 65 yrs	Ref		
	
	> 65 yrs	-3.19	[-5.06; -1.32]	< 0.001
	
	Patient gender			
	
	Female	Ref		
	
	Male	3.32	[1.51; 5.14]	< 0.001
	
	Satisfaction with life			
	
	< = 4	Ref		
	
	> 4	9.37	[7.01; 11.73]	< 0.001
	
	*Random*	σ_b_(between variability)	CI95%	
	
	Facility	3.31	[2.15; 5.10]	

**Global score** n = 1772	*Fixed*			
	
	Intercept	61.43	[55.47; 67.39]	< 0.001
	
	Absenteeism	-20.24	[-36.17; -4.32]	**0.015**
	
	Hospital facility			
	
	Public	Ref		
	
	Private	-0.51	[-4.93; 3.91]	0.814
	
	Age of patient			
	
	< = 65 yrs	Ref		
	
	> 65 yrs	-3.23	[-5.20; -1.25]	0.001
	
	Patient gender			
	
	Female	Ref		
	
	Male	6.61	[1.70; 5.50]	< 0.001
	
	Satisfaction with life			
	
	< = 4	Ref		
	
	> 4	10.60	[8.11; 13.10]	< 0.001
	
	*Random*	σ_b_(between variability)	CI95%	
	
	Facility	3.09	[1.96; 4.87]	

* Confidence intervals 95%

The estimation of variance explained by the patient level was 86.8% and by the hospital level was 13.2% for the first model (MI score). The estimation of variance explained by the patient level was 84.9% and by the hospital level was 15.1% for the second model (RS score). The estimation of variance explained by the patient level was 86% and by the hospital level was 14% for the third model (overall score).

## Discussion

The results obtained from this work suggest that short-term absenteeism among nurses is significantly correlated with quality of care in terms of patient satisfaction, and in a negative manner, in particular in relation to MI and RS. These exploratory results involve a large sample from 25 hospital facilities, both public and private, and differing in size, in several French regions.

These results are interesting for several reasons. Firstly they enable confirmation of the hypothesis of interdependence between dimensions of performance underpinning the WHO-PATH model, at least for the "patient centeredness" and "human resources" dimensions. Secondly, they consolidate and widen the scope of previously published work exploring relationships between quality of care provided and the satisfaction of professionals in the workplace. Finally, these results reemphasize the need to explore human resource indicators as explicative factors for satisfaction data, in the French context too.

Factors known to affect job satisfaction are burnout, stress, lack of autonomy, or poor cohesion in the team [20-22] and these factors are linked to inadequate organisational and managerial support [23]. This has been shown in studies on "magnet hospitals", where it is good to work and good to be cared for [15]. Aiken showed a link between job satisfaction on the one hand and quality of care or patient safety on the other [24], as did Clarke [25]. The direct relationship between absenteeism and care quality was explored by Unruh [26]: this author shows that absenteeism in conjunction with a heavy workload leads to a significant increase in incidents reported. Workload does not on its own affect patient safety, but appears liable to do so when in conjunction with staff burnout [27,28]. Overworked nurses are more tired and find it harder to cope with pressures when there are extra efforts to be made. Another study was conducted by Aiken to determine the association between increased workload and care safety. Beyond a certain patient to nurse ratio, the increase of a single patient is associated with a 7% increase in the likelihood of dying within 30 days of admission [29]. Several authors have shown the existence of a relationship between burnout among nurses and patient satisfaction [12,13,24]. Thus the results of a survey among patients and nurses indicated that, in facilities that were described by professionals as having sufficient staff and in which relationships between doctors and nurses was good, patients were more inclined to report that they were satisfied with their care [13,30]. The key role of nurses in patient satisfaction appears to be more relational than technical [12,13]. Indeed, it is nurses who connect most to patients, because they take charge of day-to-day needs. They give physical care and emotional support to both patients and families.

The present study presents numerous limitations. The study protocol was not designed in order to verify the particular hypothesis explored. This possible relationship was studied because the literature contained work on the subject, and because a large number of the hospital facilities had exhaustive data available for the indicators analysed. The PATH Project didn't include a lot a adjustment variables concerning type of professionals, hospitals or patients, and adjustment has been only based on few variables. Results have to be interpreted cautiously.

Concerning satisfaction data, the mean response rate was about 40% as expected for a postal survey [9]. However, the risk of a selection bias exists due to the relatively low number of responding patient per hospital.

Further to this, indicators' definitions for absenteeism are still the subject of debate. Short-term absenteeism as studied here takes account of WHO-PATH specifications, and concerns medically motivated absences of 2-7 days. This measure of absenteeism is assumed to reflect the social atmosphere in the workplace and the implication of staff, while long-term absenteeism is more likely to be an indicator of health status and the effects of conditions in the workplace on health. It would nevertheless be interesting to take account of non-justified or motivated absences of one day, which could reflect poor atmosphere in the workplace. According to [31], short-term absenteeism is an absence of less than three days. Certain publications distinguish between "approved and non-approved" absenteeism [32]. Some calculate the days, others the hours of unplanned absence [33]. A national working group coordinated by our team is to be set up in 2011 to define a consensus on data collection for absenteeism. A further limitation in the present study is that the data collected concerned average figures for absenteeism over one semester, and they are set against patient data from a study over 2 months, for reasons of feasibility and in compliance with WHO requirements. Future studies should integrate patient and staff data over the same period. Finally, the indicators of the WHO-PATH project ultimately aim to be used by all member countries: they therefore need, beyond their necessary validity, to be simple to handle.

## Conclusion

Despite these limitations, our results constitute a starting-point for other studies, in particular in the context of growing demographic and financial pressures [29].

Future research, for which funding has been obtained by our team, will set out to study, specifically and conjointly, the link between patient satisfaction and staff absenteeism, in particular short-term absences, which could reflect atmosphere in the workplace and burnout, adjusting on more precise data such as the circumstances of the absence from work, the workload in the unit, or the characteristics of the team.

## Competing interests

The authors declare that they have no competing interests.

## Authors' contributions

LM wrote the manuscript and was involved in the twice OMS-PATH phases. EA participated in the literature review and performed the statistical analysis. CP was involved in the first phase of OMS-PATH project and has given final approval of the version to be published. STV has given final approval of the version to be published. LG has given final approval of the version to be published. AGR has given final approval of the version to be published. PM has given final approval of the version to be published. PL has made contributions to acquisition of funding and has been involved in drafting the manuscript and revising it critically for important intellectual content. All authors read and approved the final manuscript.
